# Discrimination of Transgenic Canola (*Brassica napus* L.) and their Hybrids with *B. rapa* using Vis-NIR Spectroscopy and Machine Learning Methods

**DOI:** 10.3390/ijms23010220

**Published:** 2021-12-25

**Authors:** Soo-In Sohn, Subramani Pandian, John-Lewis Zinia Zaukuu, Young-Ju Oh, Soo-Yun Park, Chae-Sun Na, Eun-Kyoung Shin, Hyeon-Jung Kang, Tae-Hun Ryu, Woo-Suk Cho, Youn-Sung Cho

**Affiliations:** 1 Department of Agricultural Biotechnology, National Institute of Agricultural Sciences, Rural Development Administration, Jeonju 54874, Korea; pandiannsp7@gmail.com (S.P.); psy22@korea.kr (S.-Y.P.); novis7@korea.kr (E.-K.S.); happykorean@korea.kr (H.-J.K.); thryu@korea.kr (T.-H.R.); phyto@korea.kr (W.-S.C.); younsung@korea.kr (Y.-S.C.); 2 Department of Food Science and Technology, Kwame Nkrumah University of Science and Technology (KNUST), Kumasi AK-039-5028, Ghana; izaukuu@yahoo.com; 3 Institute for Future Environmental Ecology Co., Ltd., Jeonju 54883, Korea; 50joo@hanmail.net; 4 Seed Conservation Research Division, Baekdudewgan National Arboretum, Bonghwa 36209, Korea; chaesun.na@kiam.or.kr

**Keywords:** *Brassica rapa*, transgenic canola, GM detection, Vis-NIR spectroscopy, chemometrics, machine learning

## Abstract

In recent years, the rapid development of genetically modified (GM) technology has raised concerns about the safety of GM crops and foods for human health and the ecological environment. Gene flow from GM crops to other crops, especially in the Brassicaceae family, might pose a threat to the environment due to their weediness. Hence, finding reliable, quick, and low-cost methods to detect and monitor the presence of GM crops and crop products is important. In this study, we used visible near-infrared (Vis-NIR) spectroscopy for the effective discrimination of GM and non-GM *Brassica napus, B. rapa*, and F1 hybrids (*B. rapa* X GM *B. napus*). Initially, Vis-NIR spectra were collected from the plants, and the spectra were preprocessed. A combination of different preprocessing methods (four methods) and various modeling approaches (eight methods) was used for effective discrimination. Among the different combinations, the Savitzky-Golay and Support Vector Machine combination was found to be an optimal model in the discrimination of GM, non-GM, and hybrid plants with the highest accuracy rate (100%). The use of a Convolutional Neural Network with Normalization resulted in 98.9%. The same higher accuracy was found in the use of Gradient Boosted Trees and Fast Large Margin approaches. Later, phenolic acid concentration among the different plants was assessed using GC-MS analysis. Partial least squares regression analysis of Vis-NIR spectra and biochemical characteristics showed significant correlations in their respective changes. The results showed that handheld Vis-NIR spectroscopy combined with chemometric analyses could be used for the effective discrimination of GM and non-GM *B. napus, B. rapa*, and F1 hybrids. Biochemical composition analysis can also be combined with the Vis-NIR spectra for efficient discrimination.

## 1. Introduction

Oilseed rape (*Brassica napus* L.), also known as canola, is one of the most important oil crops, belongs to the Brassicaceae family which has 338 genera and 3709 species [1]. It produces 75 million tonnes per year of oil globally, among which approximately 60% of rapeseed oil is used for food, 38% for industrial uses, and 3% for feed [2]. *B. napus* (AACC, 2n = 38) originated by natural hybridization between two diploid progenitors, *B. rapa* (AA, 2n = 20) and *B. oleracea* (CC, 2n = 18) in the Mediterranean region about 7500 years ago [3,4]. Introgression of genes from *B. rapa* and synthetic materials produced by artificial crossing between the two diploid progenitors have broadened the genetic pool of *B. napus* [5,6]. Since it is closely related to several weeds and wild species and produces a large amount of pollen, when it has favorable conditions, it attains a significant degree of outcrossing (20–40%) [7,8]. Several studies have reported on the hybridization of *B. napus* with close relative species, among which *B. rapa* is the most common [8,9].

In the last three decades, GM technologies have introduced various novel characteristics into *B. napus* including increased oil content [10], drought resistance [11], herbicide resistance [12], and the production of bioactive compounds [13]. Farmers have adopted transgenic canola for its potential advantages, but the coexistence of transformants and nontransformants poses a threat to the inserted transgene spreading [14]. *B. napus* can grow on both wasteland and agricultural fields, and may develop feral wild populations that can serve as pollen donors and acceptors [15,16]. The introduction of GMOs in agricultural and food markets should be accompanied by a regulatory body to monitor the presence and quantity of GMOs. Nowadays, there are a lot of analytical methods for determining, characterization, and verifying GMOs in crops and foods. There ae DNA-based methods like Southern blot, qualitative and quantitative PCR, and real-time PCR, and protein-based methods like Western blot, ELISA, and lateral flow strip [17,18]. Overall, GMO detection approaches based on DNA and proteins are flexible, sensitive, and accurate. Nevertheless, these methods are laborious, expensive, time-consuming and require highly qualified professionals. Conversely, spectroscopy methods are nondestructive, synchronous, and involve consistent detection instruments that are environmentally benign, quick (˂1 min), low-cost, and simple to use without requiring complicated sample preparation [17]. 

The use of near-infrared (NIR) and visible NIR (Vis-NIR) spectroscopy combined with chemometric analyses has resulted in effective discrimination of GMOs in agro-food markets [18]. Vis-NIR spectroscopy is most common in the detection of GMOs used with the spectral range of 350–2500 nm (Visible (350–780) and NIR (780–2500)) overlapping with the optical radiation range (100–1000 nm) [18,19]. It works on the principle of identifying the relative proportions of C-H, N-H, and O-H bonds in organic molecules. Detection of GMOs using Vis-NIR spectroscopy is not based on the detection of changes in DNA or single proteins but on identifying the changes in structural changes due to the genotype changes caused by the introduction of transgenes for target traits [17]. Previously, several research projects were successful in using Vis-NIR spectroscopy and chemometric approaches for the effective discrimination of GM crops and foods [18,20,21,22]. Further, it is important to assess the biochemical compositional changes in the transgenic plants [23]. This can provide a correlation between spectral data prediction and the chemical composition [24]. Hence, in the present study, we aimed to explore the feasibility of effective discrimination between GM and non-GM *B. napus*, and their hybrids with *B. rapa* (*B. rapa* X GM *B. napus*), by using Vis-NIR spectroscopy in combination with different preprocessing and machine learning methods and assessing the phenolic compounds using GC-MS analysis.

## 2. Results 

### 2.1. Diffuse Reflectance Spectroscopic Analysis and Preprocessing

**Figure 1:**
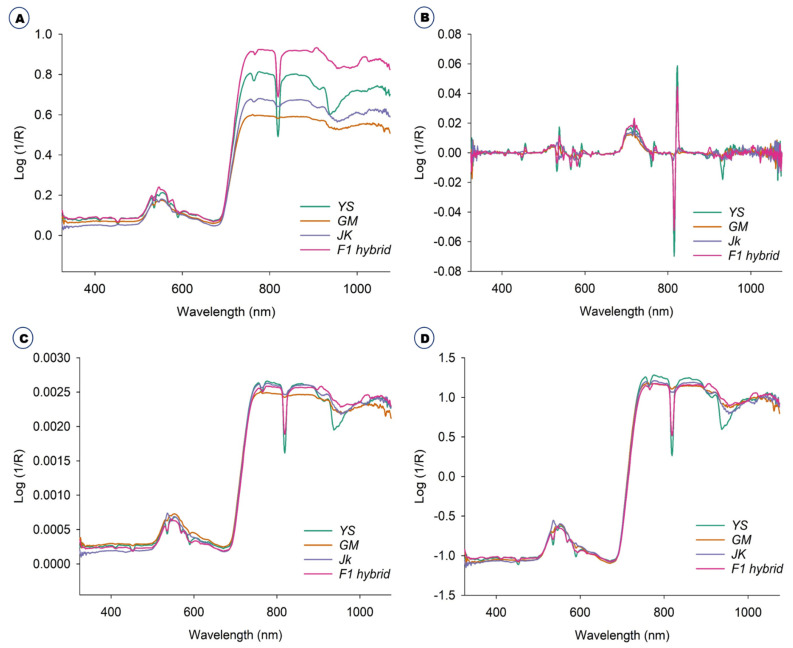
Average spectra obtained from all the plants. (**A**) Raw spectra. (**B**) Savitzky-Golay. (**C**) Normalization. (**D**) Standard Normal Variate.

**Figure 2:**
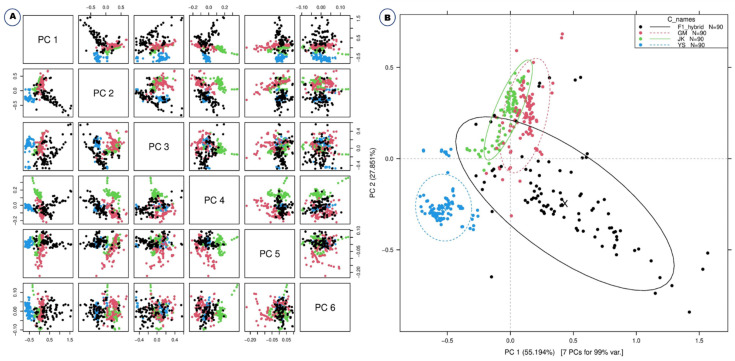
Principal Component Analysis (PCA) paired plot (**A**) and PC1 vs PC2 plot (**B**) for the visualization of *B. napus*, GM *B. napus*, *B. rapa* and F1 hybrids.

The original raw spectra of the *B. napus*, GM *B. napus*, *B. rapa* and the F1 hybrids were collected in the green house. The original raw spectra were those that had not been preprocessed in any way and the average raw spectra are shown in Figure 1A. Further, the spectra from all the plants were preprocessed with three different methods, namely, Savitzky-Golay smoothing filter (21-points), Normalization, Standard Normal Variate and averaged, as shown in Figure 1B and Figure 2C,D, respectively. 

There were differences in spectral reflectance but the majority of the spectra acquired from the four plants followed a similar pattern. The average reflectance difference between GM and non-GM *B. napus*, *B. rapa*, and F1 hybrids are assumed to be due to changes in hundreds of physicochemical constituents. The average spectra for all the plants, raw and preprocessed, with three different methods, namely, Savitzky-Golay, smoothing filter (21-points), Normalization, and Standard Normal Variate, effectively visualized the differences (Figure 1). From the PCA-paired plot from PC1 to PC 6 (Figure 2A), all the different PCs showed some pattern of separation for the different samples, but PC1 vs. PC2 showed the greatest visual differences as shown in Figure 2B. Therefore, outlier detection was performed using these two PCs before commencing preprocessing for the machine learning classification methods. In PC1 and PC2 (Figure 2B) the *B. napus* plants could be clearly separated from the others. 

### 2.2. Machine Learning Classification Methods 

**Figure 3:**
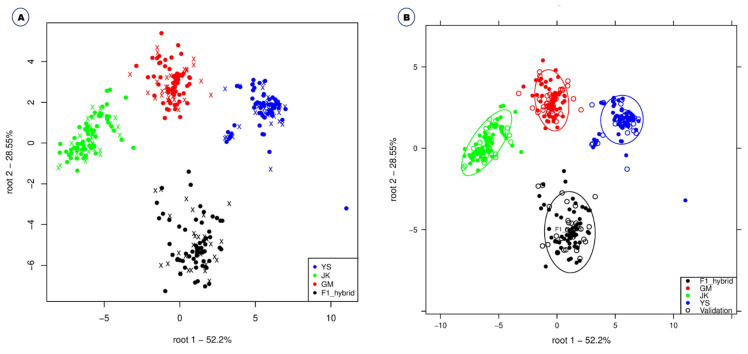
Linear Discriminant Analysis for the effective discrimination of *B. napus*, GM *B. napus*, *B. rapa* and F1 hybrids shown without confidence circles (A) and with confidence circle (B).

Convolutional neural network (deep learning), linear discriminant analysis, gradient boosted trees, support vector machine, random forest, fast large margin, generalized linear model, and naive beyes were applied to the original spectral data and preprocessed spectra using normalization, standard normal variate (SNV), and Savitzky-Golay. The classification accuracy of various machine learning approaches combined with different preprocessing methods was calculated to identify the precise method for the discrimination of GM and non-GM *B. napus*, *B. rapa* and F1 hybrids (*B. rapa* X GM *B. napus*). This ranged from 70.5% to 100% based on the preprocessing and models applied to the spectra (Table 1). Among the different modelling approaches, Support Vector Machine, Linear Discriminant Analysis and Fast Large Margin were found to have higher accuracy in combination with different preprocessing methods (Savitzky-Golay/Support Vector Machine-100%, and Savitzky-Golay/Linear Discriminant Analysis–99.8%) (Table 1). In this study, normalization yielded the least performance accuracy method among the tested preprocessing methods (Table 1). Gradient Boosted Trees and Linear Discriminant analysis accuracies were 97.3% and 98.6%, respectively, for normalization, whereas Naive Bayes and Fast Large Margin accuracies were 74.2% and 72.2%, respectively. With Savitzky-Golay preprocessing, the accuracies of Support Vector Machine and Generalized Linear Model were 100% and 97.9%, respectively, while Naive Bayes and Random Forest were 87.5% and 89%, respectively. In the case of Standard Normal Variate preprocessing, Support Vector Machine showed 98.4% accuracy, Fast Large Margin 96.2%, Generalized Linear Model 90.3%, and Naive Bayes 81.2% (Table 1). Effective discrimination of *B. napus*, GM *B. napus*, *B. rapa* and F1 hybrids using Linear Discriminant Analysis are shown in Figure 3.

The efficiency of multiple preprocessing and machine learning methods on spectral datasets obtained from the assessed plants was statistically analyzed (Table 2). After cross-validation, the mean percentage of classification accuracy of each machine learning method in combination with various preprocessing methods revealed the significance of modeling for the discrimination of GM and non-GM *B. napus*, *B. rapa* and F1 hybrids (Table 2). Statistical analysis by ANOVA (Table 3), showed the sum of square and mean sum of square values of different preprocessing and machine learning methods with statistical significance at *p* ≤ 0.05.With a combination of preprocessing and different machine learning methods used together, there was no significance with *p* ≥ 0.05 (*p*-value of 0.0925). The confusion matrix shows the degree of error in the classification of the assessed plants, which confirms that Savitzky-Golay combined with Support Vector Machine was the most effective method for the classification (Table 4).

### 2.3. Phenolic Acid Composition Analysis

Initially, the contents of total phenolic acids, including methanol-soluble and methanol-insoluble phenolic acids in plant samples of *B. napus*, GM *B. napus*, *B. rapa* and the F1 hybrids, were analyzed using GC-MS analysis. Table 5 summarizes the content of each identified compound in the GM and non-GM *B. napus*, *B. rapa* and the F1 hybrids. 

**Figure 4:**
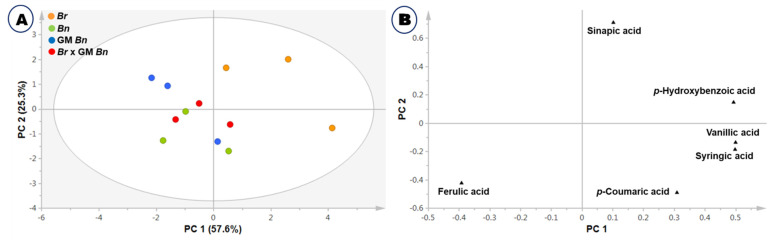
Score (**A**) and loading (**B**) plots of principal components 1 and 2 of the PCA results obtained from data on six total phenolic acids of four varieties.

The major compounds assessed, i.e., p-hydoxybenzoic acid, vanillic acid, syringic acid, *p*-coumaric acid, ferulic acid and sinapic acid, were found in different concentrations among the assessed plants. Among the compounds, ferulic acid and sinapic acid were the most abundant compounds in the *Brassica* Sp. Ferulic acid was a little lower in *B. rapa* than in *B. napus* and F1 hybrids, whereas sinapic acid was found to be higher in *B. rapa*. Similar results were obtained from the biplot of PCA in which *B. rapa*. was separated from the other plants, *B. napus*, GM *B. napus*, and the F1 hybrids (Figure 4A). The loading plot indicated that ferulic acid was lower in *B. rapa* than in other species and hybrids (Figure 4B).

### 2.4. Partial Least Squares Regression (PLSR) Prediction of Phenolic Compounds 

**Figure 5:**
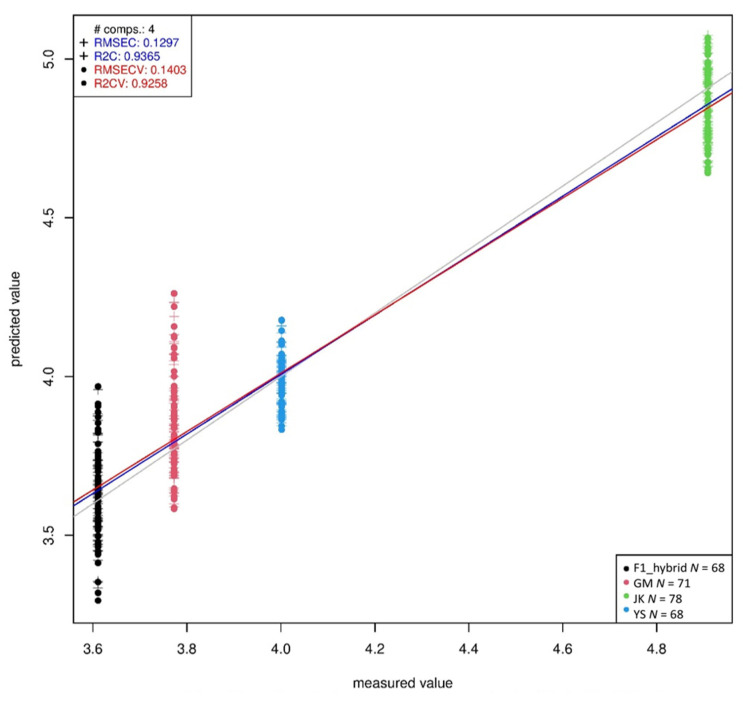
Partial least squares regression (PLSR) prediction of vanillic acid in all the plants.

Table 6 shows the PLSR prediction of phenolic compounds in all the plants. *p*-hydroxybenzoic acid, vanillic acid, syringic acid, *p*-coumaric acid, ferulic acid and sinapic acid could all be predicted with coefficients of determination after cross-validation (R2CV) above 0.89 and root mean square error after cross-validation (RMSECV) below 64.34 ug/g. Only ferulic acid and sinapic acid had high RMSECV, and R2CV’s higher than 0.89. The results prove that all the measured phenolic compounds could be predicted with high accuracy using Vis-NIR spectroscopy. Among the different compounds, vanillic acid could be predicted with the highest R2CV of 0.93 (Figure 5) and the lowest RMSECV of 0.14. 

## 3. Discussion

Physical qualities have a significant impact on product characteristics [25]. In this study, the morphological changes among *B. napus*, GM *B. napus*, *B. rapa* and F1 hybrids showed the variations in appearance. The F1 hybrids were found to have the structure of both *B. napus* and *B. rapa*. Vis-NIR spectroscopy is generally used for studying species discrimination of different plants and compositional changes of agricultural and food products [26,27]. The raw Vis-NIR spectra obtained by the handheld spectrophotometer cannot to be directly used because the number of spectra was high and the spectra were noisy [18,28]. Spectral data are mainly preprocessed to remove systemic noise to highlight the differences across the samples [18]. Utilization of different preprocessing methods simultaneously helps to achieve a different level of classification accuracy and provides an opportunity to find the best preprocessing method for a particular sample [18]. The selection of an optimum preprocessing method is difficult, since multiple different mathematical transformations are used, and different preprocessing methods provide different prediction results [29]. Generally, Vis-NIR spectra provide information on the chemical composition and physical state of the particular material, which provides structural information on the chemical functional groups of the molecules that constitute the molecular fingerprint of the sample [30,31]. Some characteristic peaks can be observed around 500–600 nm, the spectral range often being attributed to the presence of chlorophyll [32]; peaks also occur around 800 nm. However, based simply on spectral reflectance, it is difficult to distinguish these samples. Therefore, it is necessary to use principal component analysis for effective classification using Vis-NIR spectroscopy in combination with advanced chemometrics methods. For the selection of ideal preprocessing methods for spectral data, the analysis should be done with several combinations of preprocessing, statistical and modelling methods, depending on the objective of the study. Discrimination accuracy can be improved differently depending on each method of preprocessing treatment [33]. 

The use of multiple modelling approaches in combination with different preprocessing methods resulted in the discrimination of GM and non-GM *B. napus*, *B. rapa* and F1 hybrids with different classification accuracy. Previously, several studies used a combination of NIR spectroscopy and multiple machine learning/chemometric methods for the effective discrimination of GM and non-GM crops with high classification accuracies [18,22,31,34]. Higher classification accuracy was found in the combination of Savitzky-Golay and Support Vector Machine methods. There are two known advantages of using derivatives of spectra: (1) increased resolution of overlapping peaks and reduced baseline variations, and (2) more effective modeling and testing than with the original spectra [35]. Among eight different chemometrics methods used, Support Vector Machine and Linear Discriminant Analysis were found to have the highest classification accuracy. SVM is a binary classification technique that is designed to solve a classification problem and is based on statistical learning theory. It has been shown to be an effective method for nonlinear classification, multivariate function estimation, and nonlinear regression [36,37]. Considering the LDA results, LDA grouped the plants on the basis of GM and non-GM *B. napus*, *B. rapa* and the F1 hybrids separately. The Support Vector Machine and Linear Discriminant Analysis methods were found to be more effective as compared to any other chemometric method. However, the ranking of the algorithms may not be accurate due to information leakage among different machine learning algorithms. Since the spectral data from the four different plant groups were simultaneously used for the study, the possibility of information leakage was quite high. However, the main outcome of the study is a novel, rapid method of discrimination of GM and non-GM *B. napus* and their interspecific hybrids (*B. rapa* X *B. napus*). Statistical analysis revealed the results were accurate and significant. ANOVA showed the ability of preprocessing methods and models to predict with a *p* value of *p* ≤ 0.05. Similar trends were also witnessed by Sohn et al. [38] in studying the six different *Amaranthus* sp. in the fields using Vis-NIR spectroscopy coupled with modelling methods. 

*Brassica* crops are generally high in polyphenols, but the composition of phenolic compounds varies greatly between species and even between crops of the same species [39]. Flavonoids (mostly flavonols, but also anthocyanins) and hydroxycinnamic acids are the most common polyphenols found in *Brassica* sp. [40]. Polyphenolic compounds are essential components of a healthy diet. It has also been reported that they possess medicinal properties [41]. In general, as compared to other *Brassica* sp. *B. napus* has a higher level of phenolic compounds, especially ferulic acid and sinapic acid derivatives [42,43]. To confirm the validity of the NIR spectroscopy model, it is imperative to analyze the regression coefficient plots of the PLS models to check that the key wavelengths of the model are related to the spectroscopic signal of the interested constituent molecule [44,45]. Hence, the GC-MS analysis and the Vis-NIR Spectra were correlated with PLSR methods, and GM and non-GM crops were discriminated based on the polyphenolic compounds. The RMSECV levels of three hydroxybenzoic acids (*p*-hydroxybenzoic, vanillic, and syringic acids) analyzed in this study were much lower compared to the three hydroxycinnamic acids (*p*-coumaric acid, ferulic acid and sinapic acid). Recently, Peiris et al. [24] studied the discrimination of sorghum lines using NIR spectroscopy with different modelling methods and also the starch and protein content among the selected lines. They found that regression analysis resulted in the discrimination of lines according to seed starch contents. 

## 4. Materials and Methods

### 4.1. Plant Materials

**Figure 6:**
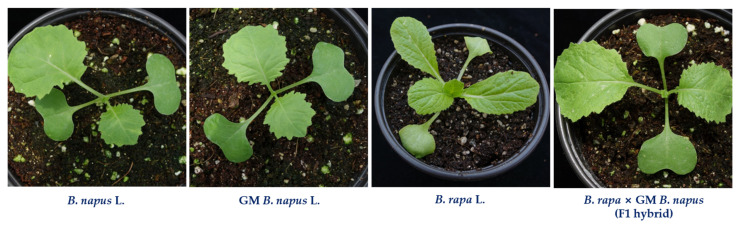
Representative photos of plants selected for the spectral analysis.

The seeds of the plants used in the study, such as *B. napus* L. ‘Youngsan’ and *B. rapa* L. ssp. *pekinensis* ‘Jangkang’ were obtained from the National Agrobiodiversity Center, Jeonju, Korea. GM *B. napus* (Youngsan) seeds with CAMV 35S-regulated *bar* gene and an early flowering gene (*BrAGL20*) were kindly provided by Yeon-Hee Lee, National Institute of Agricultural Sciences, Jeonju, Korea. For hybrid preparation, artificial hand pollination was done with *B. rapa* and *GM B. napus* and the seeds of F1 hybrids (*B. rapa* X GM *B. napus*) were used for further studies [8]. The hybrids were confirmed through a survival assay after 0.3% Bastar treatment, the phenotype of the hybrids, and polymerase chain reaction with partial 35S promoter and *BrAGL20* [8]. All the plants were grown in soil cups (Figure 6) and maintained in a controlled environment. This study was performed from May to July 2020 in the greenhouse of the National Institute of Agricultural Sciences, Jeonju, Korea. 

### 4.2. Spectral Measurement and Preprocessing

Vis-NIR diffuse reflectance spectra were collected with a handheld integrated portable spectral analyzer (FieldSpec^®^ HandHeld 2, ASD Inc., Longmont, CO, USA), working in reflectance mode (log/R) in the range of 325–1075 nm with stepping of 1.5 nm. The spectra were measured on the adaxial surface of the fully expanded leaves, which can easily capture the light. Three spectra were obtained from various parts of the leaf blade of fifty plants in each group. A total of 150 (3 × 50 = 150) spectra were collected from each group and used for further analysis. During each spectral acquisition, the Vis-NIR device’s optical window was put in direct contact with the leaf’s surface, ensuring that the sensor window was completely covered, according to the Sohn et al. [38]. Background signals appeared in the raw spectra of samples due to system parameters and environmental noise. To minimize spectral noise and improve effective information, different data preprocessing methods were employed, namely raw spectra, normalization, Savitzky-Golay, and Standard Normal Variate, which can reduce the noise and improve the accuracy of modelling approaches. The computations on preprocessing were done with Unscrambler^®^ X software, version 10.5.1 (CAMO ASA, Oslo, Norway).

### 4.3. Modelling Methods and Statistical Analysis

For the effective visualization, principal component analysis (PCA) was used to analyze patterns and variances in the dataset. This was to detect and remove outliers before developing the classification models. For the effective discrimination of spectral data, several machine learning methods were used. The modelling was performed with the software package RapidMiner studios Version 9.0.002 (Rapidminer, Inc., Boston, MA, USA). In the study, we used seven classification methods, namely, Linear Discriminant Analysis, Convolutional Neural Network (deep learning), Gradient Boosted Trees, Support Vector Machine, Random Forest, Generalized Linear Model, Fast Large Margin, and Naive Bayes to find the best modeling approach with higher classification accuracy. For each of the algorithms, the inputs were provided as the data points of the spectra and the classes were the identification labels of *B. napus*, GM *B. napus*, *B. rapa* and F1 hybrid (*B. rapa* X GM *B. napus*). The metaparameters were tuned according to the Sohn et al. [38] and Abdeni et al. [46] for the effective use of the machine learning methods through RapidMiner software package. The classification accuracy of the various machine learning approaches combined with different preprocessing methods were calculated for identifying the precise method for the discrimination of GM and non-GM *B. napus, B. rapa* and F1 hybrid between *B. rapa* and GM *B. napus.*

One-way analysis of variance (ANOVA) was performed when comparing means for testing the influence of the application of a scatter correction method, the eight classification algorithms, and the interaction of the two precious factors (preprocessing and machine learning methods). As a mean comparison method, Tukey’s range test was used at a significance level of *p ≤ 0.05*.

### 4.4. Assessment of Phenolic Acid Contents

To compare the spectral differences and chemical composition of GM and non-GM plants we assessed phenolic acid compounds using GC-MS analysis. The leaves of all the plants were collected as three biological replicates and then freeze-dried at 80 °C for at least 72 h and ground into a fine powder using a planetary mono mill (Pulverisette 6; Fritsch, Idar-Oberstein, Germany). The powder was stored at −80 °C until analysis. Methanol-soluble and methanol -insoluble phenolic acids were extracted according to the procedure described by Park et al. [47]. The powdered samples (0.01 g) were extracted by incubating at 30 °C for 10 min with 1 mL of 85% methanol containing 2 g/L butylated hydroxyanisole. After centrifugation at 13,000 rpm for 10 min at 4 °C, the supernatant and residue were analyzed to determine the quantities of soluble and insoluble phenolic acids, respectively. Hydrolysis was conducted with 1 mL 5 N NaOH at 30 °C under nitrogen gas for 4 h. All mixtures were extracted with ethyl acetate and evaporated. After derivatization by using pyridine and *N*-(*tert*-butyldimethylsilyl)-*N*-methyltrifluroacetamide with 1% *tert*-butyldimethylchlorosilane, sample (1 µL) was injected into a 7890A gas chromatograph (Agilent, Atlanta, GA, USA) with a split ratio of 10, and separated on a 30 m × 0.25-mm i.d. fused silica capillary column coated with 0.25-µm CP-SIL 8 CB low bleed (Varian Inc., Palo Alto, CA, USA). The column effluent was introduced into a Pegasus HT TOF mass spectrometer (LECO, St. Joseph, MI, USA). The detailed condition of GC-TOFMS was followed as described previously [47]. Partial least squares regression (PLSR) was used to develop models to regress on the concentrations of *p-*hydroxybenzoic acid, vanillic acid, syringic acid, *p*-coumaric acid, ferulic acid and sinapic acid in all the samples. For all predictions, the dataset was divided into calibration and validation once again, and k-fold cross-validation was used to test the predictive significance of the models. The statistical parameters used to evaluate the performance of the PLSR models were the root mean square error of calibration (RMSEC) and the coefficient of determination (R2C); in cross-validation (RMSECV, R2CV). The optimum number of latent variables was determined based on the minimum RMSECV to minimize the probability of over fitting.

## 5. Conclusions

In conclusion, Vis-NIR spectroscopy coupled with machine learning methods effectively discriminated between *B. napus* and GM *B. napus,* as well as *B. rapa* and the F1 hybrids (*B. rapa* X GM *B. napus*). Among the different combinations of preprocessing and machine learning methods, the combination of Savitzky-Golay and Support Vector Machine was found to be the most effective method, with 100% classification accuracy. The correct classification accuracy of the validation tests was achieved at 100% in a spectral range of 325–1075 nm. Further, GC-MS analysis-based phenolic acid measurements and PLSR analysis showed that the results were significantly correlated with Vis-NIR spectroscopy-based discrimination of GM and non-GM *B. napus, B. rapa* and F1 hybrids. Thus, it is suggested that this nondestructive technology can be used in the field for the rapid detection of unintentional releases of GM crops and their hybrids into the environment, and for effective management. 

## Figures and Tables

**Table 1:** Classification accuracy of the combinations of preprocessing and model for reflectance spectra from *B. napus*, GM *B. napus*, *B. rapa* and F1 hybrids.

S. No	Model	Preprocessing	Average Accuracy (%)	Run Time (ms)
1.	Linear Discriminant Analysis	Raw spectra	78.3	-
		Normalization	98.6	-
		Standard Normal Variate	98.6	-
		Savitzky-Golay	99.8	-
2.	Support Vector Machine	Raw spectra	98.4	21,417
		Normalization	79.6	41,166
		Standard Normal Variate	98.4	22,074
		Savitzky-Golay	100.0	30,556
3.	Generalized Linear Model	Raw spectra	85.4	32,905
		Normalization	87.1	19,854
		Standard Normal Variate	90.3	26,768
		Savitzky-Golay	97.9	14,038
4.	Gradient Boosted Trees	Raw spectra	95.2	841,966
		Normalization	97.3	790,162
		Standard Normal Variate	97.3	988,233
		Savitzky-Golay	98.9	990,738
5.	Naive Bayes	Raw spectra	70.5	6546
		Normalization	74.2	6535
		Standard Normal Variate	81.2	6210
		Savitzky-Golay	91.4	6661
6.	Fast Large Margin	Raw spectra	93.6	37,002
		Normalization	71.2	38,845
		Standard Normal Variate	96.2	37,597
		Savitzky-Golay	98.9	17,611
7.		Raw spectra	79.0	31,558
	Random Forest	Normalization	86.6	30,336
		Standard Normal Variate	90.9	31,411
		Savitzky-Golay	91.4	31,590
8.	Convolutional Neural Network (Deep Learning)	Raw spectra	91.4	7529
		Normalization	98.9	7123
		Standard Normal Variate	97.9	5850
		Savitzky-Golay	96.8	5450

**Table 2:** Means of percentage of classification accuracy of different preprocessing and different classification model using reflectance spectra.

Model	Species Accuracy (% ± SE)				
	Raw Spectra	Normalization	Savitzky-Golay	SNV	Significance
Naive Bayes	74.2 ± 9.5	74.5 ± 3.3 ^b^	91.8 ± 3.1	82.7 ± 4.9	ns
Generalized Linear Model	86.7 ± 3.7	87.2 ± 2 ^ab^	97.3 ± 1.5	91.3 ± 6.3	ns
Fast Large Margin	94.1 ± 4.4 ^A^	73.1 ± 4.4 ^Bb^	99.2 ± 0.8 ^A^	96.3 ± 3 ^A^	**
Convolutional Neural Network	92.8 ± 3.5	99.2 ± 0.8 a	96.9 ± 3.1	98 ± 1.2	ns
Gradient Boosted Trees	76.1 ± 12.4	85.6 ± 6.4 ^ab^	85.2 ± 6.3	59.6 ± 22	ns
Random Forest	80.8 ± 6	87.2 ± 2.3 ^ab^	92.9 ± 3.5	91.5 ± 3.3	ns
Support Vector Machine	98.4 ± 1.6 ^A^	80 ± 3.6 ^Bb^	100 ± 0 ^A^	98.3 ± 1.7 ^A^	**
significance	ns	**	ns	ns	

ns; not significant, ** significant with the *p ≤* 0.05. Different alphabetical small and capital letters show the significance of the value in the order of column and row respectively.

**Table 3:** Analysis of variance of percentage of correctly classified *B. napus*, GM *B. napus*, *B. rapa* and F1 hybrids from four different preprocessing and four different classification model using reflectance spectra.

Source	df	SS	MS	F-Value	*p*-Value
Preprocessing (P)	3	0.186074	0.062025	4.07	0.0095
Model (M)	6	0.494012	0.082335	5.4	<0.0001
P × M	18	0.426077	0.023671	1.55	0.0925
Error	84	1.280539	0.015245		
Total	111	2.386702			

df: degree of freedom. SS: sum of squares. MS: mean sum of squares.

**Table 4:** Confusion matrix of species discrimination using better performing combinations of preprocessing methods and models.

**Savitzky-Golay/** **SVM**	**Classified as**				**Average Accuracy (%)**
	*B. napus*	*B. rapa*	GM *B. napus*	F1 hybrid	
*B. napus*	43	0	0	0	100
GM *B. napus*	0	42	0	0	100
*B. rapa*	0	0	44	0	100
F1 hybrid	0	0	0	56	100
Class recall (%)	100	100	100	100	
**Normalize/** **Convolutional N** **eural N** **etwork**	**Classified as**				**Average Accuracy (%)**
	*B. napus*	*B. rapa*	GM *B. napus*	F1 hybrid	
*B. napus*	42	0	0	0	100
GM *B. napus*	0	44	0	0	100
*B. rapa*	0	0	40	0	100
F1 hybrid	0	0	2	58	96.67
Class recall (%)	100	100	95.24	100	
**Savitzky-Golay/** **Fast Large Margin**	**Classified as**				**Average Accuracy (%)**
	*B. napus*	*B. rapa*	GM *B. napus*	F1 hybrid	
*B. napus*	42	0	0	0	100
GM *B. napus*	0	44	0	0	100
*B. rapa*	0	0	40	0	100
F1 hybrid	0	0	2	58	96.67
Class recall (%)	100	100	95.24	100	

**Table 5:** Total phenolic acid composition analysis using GC-MS.

S. No	Phenolic Acids	*B. napus* L. (Youngsan) (ug/g ± SD)			GM *B. napus* L. (TG#39) (ug/g ± SD)			*B. rapa* L. (Jangang) (ug/g ± SD)			*B. rapa* X GM *B. napus* (F1 hybrid) (ug/g ± SD)		
		Soluble	Insoluble	Total	Soluble	Insoluble	Total	Soluble	Insoluble	Total	Soluble	Insoluble	Total
1	*p*-hydroxybenzoic acid	2.2 ± 0.4	1.1 ± 0.3	3.3 ± 0.6	2.3 ± 0.1	0.9 ± 0.1	3.1 ± 0.3	4.1 ± 0.7	1.3 ± 0.3	5.4 ± 1.0	2.2 ± 0.4	1.3 ± 0.8	3.5 ± 0.8
2	vanillic acid	3.0 ± 0.6	1.0 ± 0.2	4.0 ± 0.7	2.7 ± 0.3	1.1 ± 0.2	3.8 ± 0.5	3.9 ± 0.8	1.0 ± 0.2	4.9 ± 0.9	2.6 ± 0.6	1.0 ± 0.1	3.6 ± 0.5
3	syringic acid	0.3 ± 0.2	0.3 ± 0.2	0.6 ± 0.3	0.3 ± 0.2	0.3 ± 0.2	0.6 ± 0.3	0.6 ± 0.3	0.4 ± 0.3	1.0 ± 0.4	0.3 ± 0.2	0.3 ± 0.04	0.6 ± 0.2
4	*p*-coumaric acid	56.1 ± 14.4	6.9 ± 0.7	63.0 ± 13.8	28.1 ± 17.1	5.5 ± 0.9	33.7 ± 18.0	49.9 ± 15.7	12.7 ± 1.5	62.6 ± 15.0	56.2 ± 6.4	6.1 ± 4.2	62.3 ± 10.5
5	ferulic acid	1498.8 ± 184.2	110.4 ± 17.6	1609.2 ± 197.5	1255.9 ± 120.6	128.1 ± 8.3	1384.0 ± 125.7	891.5 ± 51.4	49.4 ± 9.2	940.9 ± 60.5	1167.8 ± 132.1	86.3 ± 10.2	1254.1 ± 140.5
6	sinapic acid	877.3 ± 138.9	26.5 ± 4.8	903.77 ± 140.38	935.8 ± 427.3	37.2 ± 14.6	973.06 ± 441.83	1439.2 ± 518.4	35.2 ± 8.6	1474.3 ± 511.6	923.6 ± 73.0	35.2 ± 6.0	958.80 ± 78.64

**Table 6:** PLSR prediction of phenolic compounds in all plants.

Phenolic Compound	Latent Variable	R2	RMSEC (ug/g)	R2CV	RMSECV (ug/g)
*p*-hydroxybenzoic acid	4	0.93	0.26	0.91	0.28
Vanillic acid	4	0.94	0.13	0.93	0.14
Syringic acid	4	0.92	0.04	0.91	0.05
*p*-coumaric acid	4	0.91	3.68	0.89	4.03
Ferulic acid	4	0.94	58.91	0.93	64.34
Sinapic acid	4	0.94	57.89	0.93	63.64

## Data Availability

The data presented in the study are available on request from the corresponding author. The data are not available due to privacy and ethical reasons.
